# Trans-Omic Analysis Identifies the ‘PRMT1–STAT3–Integrin αVβ6 Axis’ as a Novel Therapeutic Target in Tacrolimus-Induced Chronic Nephrotoxicity

**DOI:** 10.3390/ijms262110282

**Published:** 2025-10-22

**Authors:** Sho Nishida, Tamaki Ishima, Daiki Iwami, Ryozo Nagai, Kenichi Aizawa

**Affiliations:** 1 Department of Translational Research, Clinical Research Center, Jichi Medical University Hospital, Shimotsuke 329-0498, Japan; 2 Division of Renal Surgery and Transplantation, Department of Urology, Jichi Medical University, Shimotsuke 329-0498, Japan; 3 Jichi Medical University, Shimotsuke 329-0498, Japan; 4 Clinical Pharmacology Center, Jichi Medical University Hospital, Shimotsuke 329-0498, Japan

**Keywords:** tacrolimus, trans-omics, transcriptomics, proteomics, PRMT1, integrin αVβ6, STAT3, kidney transplantation, nephrotoxicity

## Abstract

Tacrolimus-induced chronic nephrotoxicity (TACN) represents a major barrier to long-term graft survival in kidney transplantation, yet its molecular pathogenesis remains incompletely understood. We have previously reported metabolic abnormalities, including carnitine deficiency, nicotinamide adenine dinucleotide depletion, and elevated asymmetric dimethyl arginine (ADMA), in TACN. To identify upstream regulators associated with these metabolic disturbances, we conducted a comprehensive trans-omic analysis, integrating transcriptomics and proteomics of kidney tissues from male ICR mice with TACN (n = 5/group). Differentially expressed genes and proteins were subjected to functional enrichment and transcription factor binding motif analyses, followed by upstream master regulator identification using the Genome Enhancer platform. A total of 785 genes and 2472 proteins were differentially expressed, with partially discordant regulation between transcriptomic and proteomic profiles, underscoring the limitations of single-omic approaches. Upstream analysis identified protein arginine methyltransferase-1 (PRMT1) and integrins, particularly αVβ6, as potential master regulators and therapeutic targets. PRMT1 is implicated in ADMA-mediated nitric oxide inhibition and fibrosis, whereas integrin αVβ6 is associated with tubular injury and renal fibrogenesis. Notably, PRMT1 may activate STAT3, which in turn regulates integrin β6 expression, suggesting a novel PRMT1–STAT3–integrin αVβ6 axis in TACN pathogenesis. This study represents the first trans-omic approach to TACN, providing a foundation for mechanistic validation and therapeutic exploration of PRMT1 and integrins in both preclinical and clinical settings.

## 1. Introduction

Tacrolimus (TAC) (Supplemental Figure S1) [1] is an important immunosuppressant drug used in solid organ transplantation [2,3]. TAC has enabled suppression of acute rejection in kidney transplantation, leading to a significant improvement in short-term graft survival [4]. Accordingly, TAC is used in approximately 90% of immunosuppressive drug protocols worldwide in kidney transplantation [5]. However, chronic kidney disease (CKD) induced by TAC, known as Tacrolimus-induced chronic nephrotoxicity (TACN) has become a major obstacle to long-term graft survival [6,7]. TACN is defined as irreversible chronic kidney damage caused by continuous exposure to TAC [7,8]. TACN is characterized pathologically by specific arterial hyalinosis and tubular/interstitial fibrosis [9], which suggest vascular endothelial damage and tubular and interstitial injury. However, detailed pathophysiology of this chronic nephrotoxicity remains unknown, and no therapeutic approach has been developed until now [7].

Recent advances in mass spectrometry have enabled comprehensive analytical approaches such as metabolomics, transcriptomics, and proteomics, which have helped to clarify CKD pathophysiology and its primary diseases [10,11,12,13,14,15]. We have previously conducted comprehensive metabolic analyses using metabolomics to gain insights into TACN pathology. As a result, we have identified pathophysiological abnormalities such as carnitine deficiency in renal tissue [6]. We have also reported nicotinamide adenine dinucleotide (NAD^+^) deficiency and elevation of asymmetric dimethyl arginine (ADMA) in whole blood of subjects with TACN [16]. However, metabolic changes are regulated by enzymes and genes encoding proteins [17,18,19]. Therefore, to further understand the pathophysiology of TACN, it is necessary to focus on enzymes and genes that regulate metabolites. There have been several transcriptomic and proteomic analyses of renal tissue in TACN [20,21]. Demirci et al. reported on TACN pathogenesis using transcriptomics, proteomics, and phosphoproteomics [20]. Yen et al. also analyzed this pathology using transcriptomics and metabolomics [21]. However, both studies remain at the stage of comparing which genes and proteins co-vary and which pathways are enriched in their respective omics analysis. No reports have integrated transcriptomic and proteomic results to identify therapeutic target molecules in a trans-omic framework.

In this study, we adopted a trans-omic strategy by combining transcriptomic and proteomic data to identify upstream molecular regulators that connect previously reported metabolic abnormalities with molecular mechanisms underlying TACN. To achieve this, we performed in silico network analysis using the Genome Enhancer platform.

## 2. Results

### 2.1. Transcriptome Analysis and GO Analysis Functional Classification of Genes

**Figure 1:**
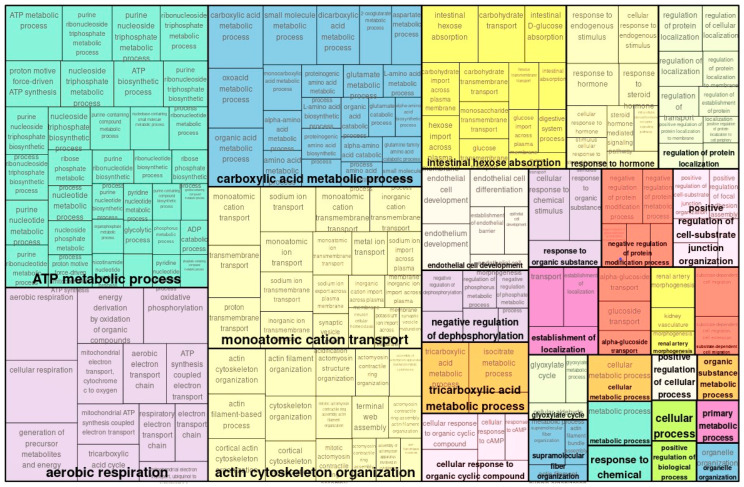
Enriched GO (biological process) map of transcriptomically upregulated genes in TAC vs. control.

**Figure 2:**
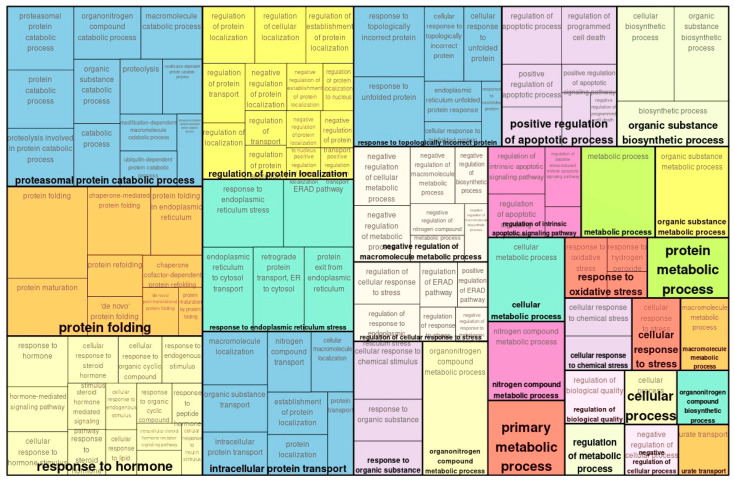
Enriched GO (biological process) of map of transcriptomically downregulated genes in TAC vs. control.

In transcriptome analysis, 931 genes were either upregulated or downregulated, with 372 significantly upregulated and 413 significantly downregulated. Totally, 785 were changed significantly. In GO analysis, genes that were up- or downregulated to a lesser degree were classified as *ATP metabolic process*, *aerobic respiration*, and *carboxylic process* (Figure 1). Downregulated gene classes included *proteasomal protein catabolic process*, *protein folding*, and *response to hormones* (Figure 2).

### 2.2. Proteome Analysis and GO Analysis of Proteins

**Figure 3:**
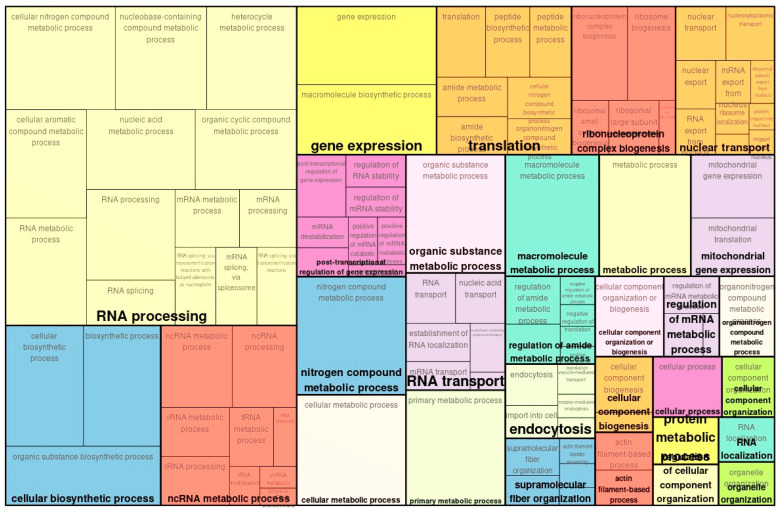
Enriched GO (biological process) of map of upregulated genes in TAC vs. controls with proteomics.

**Figure 4:**
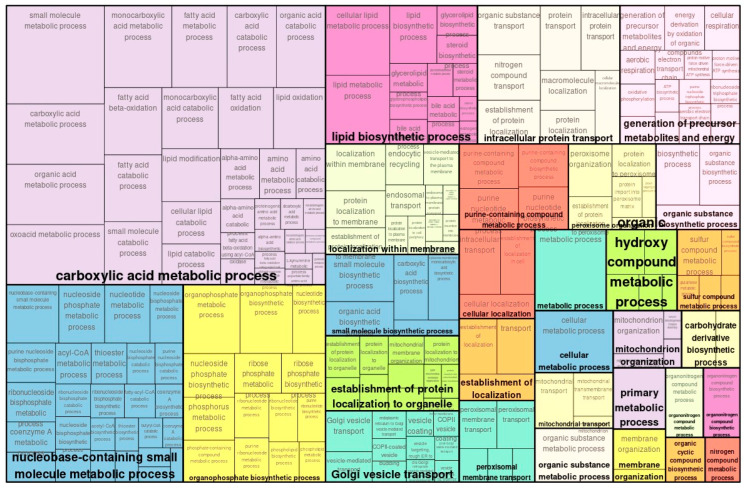
Enriched GO (biological process) of map of downregulated genes in TAC vs. controls with proteomics.

In proteome analysis, 2484 upregulated proteins were detected, of which 1373 were significant. Similarly, 2194 downregulated proteins were identified, of which 1099 were significantly downregulated. In total, 2472 proteins showed significant changes. In GO proteomic analysis, categories that were less significantly upregulated included *RNA processing*, *cellular biosynthetic process*, and *ncRNA metabolic process* (Figure 3). Categories of similarly downregulated genes in the GO analysis included the *carboxylic acid metabolic process* and *nucleobase-containing small molecule metabolic process* (Figure 4).

### 2.3. Comparison of Transcriptomic and Proteomic Expression

**Figure 5:**
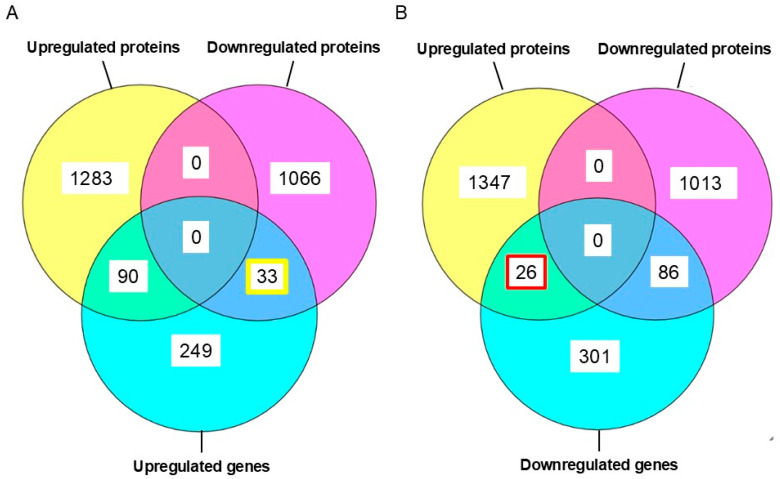
Comparison of up- and downregulated proteins with their up- and downregulated genes. (**A**) Thirty-three upregulated genes encoded downregulated proteins (yellow rectangle). (**B**) Twenty-six downregulated genes were associated with upregulated proteins (red rectangle).

A Venn diagram comparing genes and proteins detected by transcriptomic and proteomic analysis is shown in Figure 5. Proteins corresponding to genes detected in transcriptomics did not all show consistent increases or decreases in proteomic analysis. Rather, some genes showed transcriptomic increases or decreases opposite to their proteomic responses (Figure 5A,B). Among proteins corresponding to upregulated genes in transcriptomics, 33 were downregulated (Figure 5A). Among proteins corresponding to downregulated genes, 26 were upregulated (Figure 5B). Such conflicting results demonstrate the risks of attempting to understand biological phenomena with only a single type of omics (Figure 1 and Figure 4).

### 2.4. Detecting Transcription Factors

In the next step, we searched for TFBSs in regulatory regions of target genes using the transcription factor binding motif library from the TRANSFAC^®^ (Braunschweig, Germany) database. We searched for composite modules that act as potential condition-specific enhancers of target genes in their upstream regulatory regions (−1000 bp upstream of transcription start site), to identify transcription factors regulating the activity of those genes through such enhancers.

To construct the most specific composite modules, the top 300 genes that showed significant differences between the TAC and control groups were selected as input genes for the CMA algorithm. We applied the CMA [22] method to detect enhancer candidates that may function through coordinated binding of multiple TFs to regulatory regions of target genes (Supplemental Figure S2).

The resulting CMA model was then applied to calculate CMA scores for all upregulated and downregulated genes in the TAC and control groups. Then, we evaluated TFs regulating upregulated and downregulated genes with regulatory scores and YES-NO ratios. As a result, 11 TFs regulating expression of upregulated genes were identified (Table 1). The top three TFs were hepatocyte nuclear factor 4 alpha (HNF4A), signal transducer and activator of transcription 3 (STAT3), and lymphoid enhancer and activator of transcription 3 (LEF1). Nineteen TFs regulating expression of downregulated genes were also identified (Table 2). The top three TF were heat shock transcription factor 1 (HSF1), SMAD family member 3 (SMAD3), and SMAD family member 2 (SMAD2).

### 2.5. Identification of Master Regulators Through Upstream Network Analysis

Next, we identified common regulatory factors (upstream regulatory factors) among identified TF groups. Using proteomic data, we selected differentially expressed proteins involved in signaling pathways and used these proteins as a “context set” [23] in the master regulator identification algorithm. These master regulators exert significant influence on the control of intracellular signaling pathways and activate pathological processes (Table 3 and Table 4). Additionally, diagrams of intracellular regulatory signal transduction pathways for upregulation and downregulation are presented in Supplemental Figures S3 and S4. Table 3 and Table 4 show the top 10 master regulators detected in pathways of upregulated and downregulated genes, by total rank. Total rank was calculated by summing the key node score, the CMA score, and logFC data.

### 2.6. Identification of Therapeutic Target Molecules in Master Regulators

After detecting master regulators, we evaluated the druggability of master regulators potentially involved in regulation of TACN-related genes. Specifically, for the identified master regulators, we used the Human PSD™ database [24] and PASS software (PASS-2020-Standard, https://genexplain.com/pass/, accessed on 14 February 2025) [25] to evaluate their druggability. Human PSD^TM^ is the database of gene–disease–drug assignments. PASS predicts biological activity of chemical compounds based on (Q)SAR.

The druggability score represents the number of drugs that are potentially suitable to inhibit the corresponding target, either according to information extracted from the medical literature (from Human PSD^TM^ database) or according to cheminformatic predictions of compound activity against the examined target (from PASS). The master regulatory proteins detected as drug targets using Human PSD^TM^ are shown in Table 5, and the master regulatory proteins detected based on PASS are shown in Table 6. The most likely therapeutic targets for TACN based on druggability scores were integrins and protein arginine methyltransferase-1 (PRMT1).

## 3. Discussion

Metabolite analysis of TACN identified carnitine deficiency in kidney tissue and NAD^+^ deficiency in whole blood samples. These metabolic changes are associated with various enzyme genes, as well as transcription factors and master regulators. Therefore, integrating these factors is crucial to understanding the pathophysiology of TACN.

In GO analysis of the transcriptome, genes associated with enhanced glucose metabolism and energy metabolism were upregulated, suggesting that enhanced energy metabolism compensates for tissue damage. On the other hand, some discordant results were observed between the transcriptome and proteome in *carboxylic acid metabolic process*. This category was upregulated in the transcriptome (Figure 1), but downregulated in the proteome (Figure 4). Additionally, among genes and proteins detected in the transcriptome and proteome, some molecules showed differing increases and decreases despite being from the same kidney tissue. These results highlight the need to analyze the simulated temporal changes and molecular interactions between genes and proteins for each molecule.

In this multi-omic analysis, we analyzed key TFs involved in TACN. From candidate TFs, we further identified upstream master regulators in silico, based on structural features amenable to therapeutic intervention.

Using in silico analysis, integrins α and β6 were primarily identified (Table 6), consistent with previous reports [26], since these are considered clinically significant. Integrin αVβ6 occurs in small amounts in the renal tubular epithelium of normal kidneys [27], but increases in concentration when tubular damage occurs [28]. Elevated levels of αVβ6 are reported during tissue remodeling in human endothelial cells [29]. Knocking out β6 in animal models improves renal fibrosis [30], and depleting αV integrin in vivo prevents fibrosis in solid organs [31]. Inhibiting integrin αVβ6 in mouse models is associated with suppression of renal fibrosis [27,32]. Interestingly, integrin αVβ6 is elevated in kidney samples from CKD patients, and it has been reported that knocking out αVβ6 in mice suppresses macrophage migration and controls renal fibrosis [32]. These results suggest that controlling integrin αVβ6 holds potential for therapeutic intervention in TACN.

In this analysis, PRMT1 is primarily associated with ADMA synthesis through asymmetric methylation of arginine and is linked to elevated ADMA levels [33]. ADMA reportedly inhibits NOS and is associated with epithelial cell dysfunction [34]. It is elevated in patients with renal failure and is strongly associated with prognosis and cardiovascular events [35,36]. In fact, there are reports that ADMA promotes fibrosis of renal cells in vitro [37]. Since our previous report showed that ADMA in whole blood is significantly elevated in TACN mice [16], it appears that PRMT1 contributes to TACN pathophysiology through elevation of ADMA.

PRMT1 itself has been associated with chronic kidney disease in multiple studies [38,39,40]. Wang et al. reported that in vitro experiments with HK2 cells showed that TGFβ/SMAD3 signaling is mediated by PRMT1 overexpression, promoting tubular epithelial fibrosis (EMT) and leading to epithelialization of HK2 cells [38]. Additionally, several reports showed that PRMT1-mediated methylation promotes renal fibrosis, activates renal fibroblasts, and induces EMT in renal tissue from UUO mouse models [39,40]. These findings are consistent with pathological features of TACN, such as arterial hyalinization and tubular fibrosis.

To date, no report has described the relationship between the two key molecules identified as therapeutic targets in this study. However, this possibility warrants further investigation. In fetal mice, PRMT1 has been reported to methylate and activate the transcription factor STAT3 [41]. Interestingly, there are also reports indicating that STAT3 positively regulates expression of β6 in integrin αVβ6 [42,43]. In our current analysis, STAT3 is identified as a TF involved in gene changes observed in transcriptomics. These results suggest that increased PRMT1 may induce upregulation of integrin αV6.

**Figure 6:**
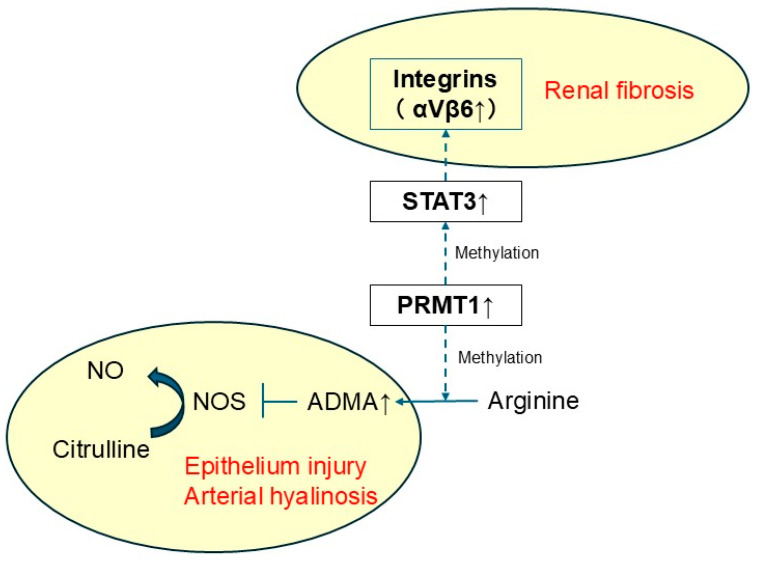
Hypothetical mechanism of TACN. The schematic depicts the relationship between TACN and therapeutic target molecules detected in this study. Integrin αVβ6 and PRMT1 are represented as therapeutic target molecules. STAT3 is detected as a key TF in this study. Solid lines indicate relationships between substrates and metabolites. Dotted lines link actions to downstream effects. ADMA: asymmetric dimethyl arginine; NO: nitric oxide; NOS: nitric oxide synthase; PRMT1: protein arginine methyltransferase-1; STAT3: signal transducer and activator of transcription 3. TF: transcription factor.

This new hypothesis, that the ‘PRMT1–STAT3–Integrin αVβ6 axis’ is the mechanism of TACN, is consistent with currently recognized clinical characteristics of TACN (Figure 6). TACN diagnosis is confirmed by graft biopsy, with specific arterial hyalinosis and tubulointerstitial fibrosis [7,8,44]. PRMT1 increases ADMA levels [33,34], suggesting vascular endothelial dysfunction in TACN. In addition, PRMT1 itself is implicated in kidney tissue injury [39]. Furthermore, integrin αVβ6 is upregulated in renal injury and promotes renal fibrosis [28,29]. These findings demonstrate that the PRMT1–STAT3–integrin αVβ6 axis presents the first comprehensive functional explanation for TACN pathophysiology.

The PRMT1–STAT3–Integrin αVβ6 axis also suggests a potential therapeutic approach to avert TAC toxicity. Bexotegrast, one of the integrin αVβ6 inhibitors, ameliorates organ damage and kidney fibrosis [45]. Framidine and GSK3368715, PRMT1 inhibitors, are also reported as potential therapeutic drugs in cardiac disorders and malignancy [46,47,48,49]. These drugs may have potential therapeutic effects against TACN, but further studies are needed for verification.

The limitations of this study are as follows. First, it employed a mouse model. Secondly, results were obtained through in silico analysis, and further validation is needed by actually inhibiting PRMT1 and integrins, especially integrin αVβ6. It is necessary to obtain similar results in human samples.

Despite being derived from an automated in silico analysis, the present results support the phenomenon identified in our previous report about TACN. Furthermore, this was the first attempt to evaluate proteomics and transcriptomics of kidney tissue in TACN. This study offers new insights into TACN. Based on this study, it is necessary validate the mechanism with human kidney samples of TACN. Interventional experiments with the TACN mouse model using therapeutic drugs for PRMT1 and integrin αVβ6 are also crucial in order to extend this study.

## 4. Materials and Methods

### 4.1. Sample Collection

The present mouse model of TACN is based on methods used in several previous reports [6,50]. Sample sizes were determined based on reports using similar animal models [6,12,16,50,51,52,53]. In brief, ten 7-week-old male ICR mice were housed separately in mouse cages (Maxi-Miser^®^ Caging System #5, Oriental Giken Corporation, Tokyo, Japan) with a 12/12 h light/dark cycle. Humidity was maintained at 40 ± 10% and 23 ± 2 °C. Mice were maintained on a low-sodium diet (0.01% sodium, CLEA Japan, Inc., Tokyo, Japan) and tap water. They were randomly separated into two groups after 7 days (n = 5 per group). Mice in the TAC group were treated with osmotic pumps (ALZET^®^ osmotic pumps 2004, Cupertino, CA, USA), and TAC (Prograf^®^, Astellas Pharma Inc., Tokyo, Japan) was administered subcutaneously at 1 mg/kg/day following previous reports [6,50]. The control group received normal saline using the same approach. There were no exclusion criteria in this study. Samples were collected after 28 days of continuous subcutaneous administration. Under isoflurane anesthesia (induction concentration: 4–5%; maintenance concentration: 2–3%), kidney tissues (20–30 mg) were collected on ice and stored at −80 °C until analysis. This animal protocol was approved by the Animal Welfare Committee of Jichi Medical University (protocol code: 23008-02, 8 November 2024).

### 4.2. Sample Preparation for Transcriptome Analysis

Tissues were dissolved in 0.5 mL of TRIzol^®^ reagent (Thermo Fisher Scientific, Waltham, MA, USA) and thoroughly crushed using the crushing rod of a BioMasher^®^ II (Nippi, Inc., Tokyo, Japan). To the resulting tissue lysate, 0.5 mL of TRizol were added, stirred well, and incubated for 5 min at room temperature. Then, 200 μL of chloroform were added to the tissue lysate, incubated for 3 min at room temperature, and centrifuged at 4 °C for 15 min. After centrifugation, the aqueous phase was fractionated for RNA purification, and protein was extracted from the remaining fraction. The upper aqueous layer was centrifuged for 10 min at 4 °C. Then, the aqueous phase was transferred to a new tube, and 20 μg of glycogen (COSMO BIO Co., Ltd., Tokyo, Japan) were added. RNA was precipitated by adding 500 μL of 2-propanol and centrifuging for 10 min at 4 °C. RNA pellets were washed with 75% ethanol and then dissolved in nuclease-free water. The concentration and quality of RNA were verified using an Agilent 2100 Bioanalyzer (Agilent Technologies, Santa Clara, CA, USA). Purified total RNA (500 ng) with an RNA integrity value of >8.7 was used for RNA library preparation according to instructions of the QuantSeq 3′mRNA-Seq Library Prep Kit FWD with UDI (Lexogen, Vienna, Austria). Libraries were amplified with 12 cycles of polymerase chain reaction. RNA libraries were sequenced using an Illumina (San Diego, CA, USA) NextSeq 500 system (75 cycles). FASTQ files were prepared with reads using bcl2fastq ver2.20 (Illumina). FASTQ sequence data were assessed using StrandNGS ver4.1 (Agilent Technologies, Santa Clara, CA, USA). After removing adapter sequences from raw reads, trimmed reads were aligned to the mm10 mouse reference genome. Expression levels of genes identified in the transcriptome were normalized and compared.

### 4.3. Sample Preparation for Proteome Analysis

Tissues were dissolved in 0.5 mL of TRIzol^®^ reagent (Thermo Fisher Scientific, Waltham, MA, USA) and thoroughly crushed using the crushing rod of a BioMasher^®^ II (Nippi, Inc., Tokyo, Japan). To the resulting tissue lysate, 0.5 mL of TRizol were added, stirred well, and incubated for 5 min at room temperature. Then, 200 µL of chloroform were added to the tissue lysate, incubated for 3 min at room temperature, and centrifuged at 4 °C for 15 min. After centrifugation, the aqueous phase was fractionated for RNA purification, and protein was extracted from the remaining fraction. Three volumes of ethanol were added to the remaining fraction and incubated for 15 min at room temperature and then centrifuged at 4 °C for 15 min. After centrifugation, precipitates were dissolved in 100 mM Tris–HCl (pH 8.0) containing 4% sodium dodecyl sulfate, 20 mM NaCl, and 10% ACN using BIORUPTOR BR-II (SONIC BIO Co., Kanagawa, Japan). Extracted proteins were quantified using Pierce™ BCA Protein Assay Kits (Thermo Fisher Scientific, Waltham, MA, USA) at 200 ng/μL. Protein purification and digestion were performed using the sample preparation (SP3) method. Tryptic digestion was performed using 1 μg Trypsin/Lys-C Mix (Promega, Madison, WI, USA) overnight at 37 °C. Protein extracts were reduced in 10 mM tris(2-carboxyethyl) phosphine and 40 mM of 2-chloroacetamide for 15 min at 80 °C, and then 5% TFA (16 μL) was added. These digests were purified using GL-Tip SDB (GL Sciences, Tokyo, Japan) according to the manufacturer’s protocol. Peptides were dissolved again in 0.1% TFA containing 0.02% DMNG and quantified using the BCA assay at 100 ng/μL.

### 4.4. LC-MS/MS

Tryptic peptides were loaded directly into the chromatograph using a 75 μm × 12 cm nanoLC nano-capillary column (Nikkyo Technos Co., Ltd., Tokyo, Japan) at 50 °C and then separated with a gradient (mobile phase A = 0.1% FA in water; B = 0.1% FA in 80% ACN) consisting of 0 min 8% B, 32 min 37% B, 38 min 75% B, and 40 min 75% B at a flow rate of 200 nL/min using an UltiMate 3000 RSLCnano LC system (Thermo Fisher Scientific). Eluted peptides were detected using a Q Exactive HF-X Hybrid Quadrupole-Orbitrap mass spectrometer (Thermo Fisher Scientific) with normal window DIA. Conditions of MS1 and MS2 were as follows.
**Condition****MS 1****MS 2***m*/*z*495–745More than 200Mass resolution30,00030,000Auto gain control target3 × 10^6^3 × 10^6^Maximum injection time55 msAutoFixed normalized collision energy-23%

### 4.5. Data Processing

Raw data were searched against an in silico-predicted spectral library using DIA-NN (version:1.9.1, https://github.com/vdemichev/DiaNN, accessed on 18 November 2024). First, the in silico-predicted spectral library was generated from the mouse protein sequence database (UniProt id UP000000589, reviewed, canonical, 21,709 entries) using DIA-NN. DIA-NN search parameters were as follows: protease, trypsin; missed cleavages, 1; peptide length range, 7–45; precursor charge range, 2–4; precursor mass range, 500–740; fragment ion *m*/*z* range, 200–1800; mass accuracy, 10 ppm; static modification, cysteine carbamidomethylation; and enabled options, “Heuristic protein interferences,” “Use isotopologues,” “MBR,” and “No shared spectra.” Additional commands were set as follows: “mass acc cal 10,” “peak translation,” and “matrix spec q”. The protein identification threshold was set at <1% for both peptide and protein false discovery rates (FDRs). Statistical calculations and Pearson correlation coefficient heatmap analysis with hierarchical clustering were performed using Perseus v1.6.15.0.

### 4.6. Data Analysis Using Genome Enhancer

#### 4.6.1. Databases Used in the Study

Transcription factor binding sites in promoters and enhancers of differentially expressed genes were analyzed using known DNA binding motifs described in the TRANSFAC^®^ library, release 2024.2 (geneXplain GmbH, Wolfenbüttel, Germany) (https://genexplain.com/transfac, accessed on 14 February 2025). The master regulator search used the TRANSPATH^®^ database (BIOBASE), release 2024.2 (geneXplain GmbH, Wolfenbüttel, Germany) (https://genexplain.com/transpath, accessed on 14 February 2025). A comprehensive signal transduction network of human cells was built with software on the basis of reactions annotated in TRANSPATH^®^. Information about drugs corresponding to identified drug targets and clinical trials references was extracted from the HumanPSD™ database, release 2024.2 (https://genexplain.com/, accessed on 14 February 2025). The Ensembl database, release Human112.38 (hg38) (https://www.ensembl.org, accessed on 14 February 2025), was used for gene ID representation, and Gene Ontology (GO) (http://geneontology.org, accessed on 14 February 2025) was used for functional classification of the studied gene set.

#### 4.6.2. Methods for Analysis of Enriched Transcription Factor Binding Sites and Composite Modules

Transcription factor binding sites in promoters and enhancers of differentially expressed genes were analyzed using known DNA binding motifs. These motifs were specified using position weight matrices (PWMs) that give weights to each nucleotide in each position of the DNA binding motif for a transcription factor or a group thereof.

We searched for transcription factor binding sites (TFBSs) that were enriched in promoters and enhancers compared to a background sequence set of promoters of genes that were not differentially regulated under conditions of the experiment. We denoted study and background sets as “Yes” and “No” sets. We considered promoter sequences of 1100 bp (−1000 to +100). The error rate in this part of the pipeline was controlled by estimating the adjusted *p*-value (using the Benjamini–Hochberg procedure) in comparison to the TFBS frequency found in randomly selected regions of the human genome (adj.*p*-value < 0.01).

We applied the Composite Module Analyst (CMA) algorithm to search for composite modules [22,54] in promoters and enhancers of the Yes and No sets. We searched for composite modules consisting of clusters of 10 TFs in a sliding window of 200–300 bp that separated sequences in the Yes and No sets (minimizing Wilcoxon *p*-value) in a statistically significant manner.

#### 4.6.3. Methods for Finding Master Regulators in Networks

We searched for master regulator molecules in signal transduction pathways upstream of identified transcription factors. The master regulator search uses a comprehensive signal transduction network of human cells, the main algorithm for which was described earlier [23,55]. The goal of the algorithm is to find nodes in the global signal transduction network that may potentially regulate the activity of a set of transcription factors found in the previous step of the analysis. Such nodes are considered the most promising drug targets, since they may switch transcriptional programs of hundreds of genes regulated by respective TFs. In our analysis, we ran the algorithm with a maximum radius of 12 steps upstream of each TF in the input set. The error rate of this algorithm is controlled by applying it 10,000 times to randomly generated sets of input transcription factors of the same set size. Z-scores and FDR values of ranks are then calculated for each potential master regulator node on the basis of such random runs (see detailed description in [56]). We controlled the error rate with an FDR threshold of 0.05.

#### 4.6.4. Methods for Analysis of Pharmaceutical Compounds

We sought the optimal combination of molecular targets (key elements of the regulatory network of the cell) that potentially interact with pharmaceutical compounds from a library of known drugs and biologically active chemical compounds, using information about known drugs from HumanPSD™ and predicting potential drugs using PASS program.

#### 4.6.5. Methods for Analysis of Known Pharmaceutical Compounds

We selected compounds from the HumanPSD™ database that have at least one target. Next, we sorted compounds using “Drug rank”, which is the sum of the following ranks:

(i) Ranking by “Target activity score” (*T-score_PSD_*);
(ii) Ranking by “Disease activity score” (*D-score_PSD_*);
(iii) Ranking by “Clinical validity score”.

The “Target activity score” (*T-score_PSD_*) is calculated as follows:

$${T-score}_{\mathrm{PSD}}=-\frac{\left|T\right|}{\left|T\right|+\omega \left(\left|AT\right|-\left|T\right|\right)}\sum_{t\in T}log10\left(\frac{rank\left(t\right)}{1+maxRank\left(T\right)}\right),$$

where *T* is the set of all targets related to compounds intersecting the input list. |*T*| is the number of elements in *T*. *AT* and |*AT*| are sets of all targets related to the compound and the number of elements in each. w is weight multiplier. *Rank(t)* is the rank of a given target, and *maxRank*(*T*) equals max(*rank*(*t*)) for all targets *t* in *T*.

We use the following formula to calculate the “Disease activity score” (*D-score_PSD_*):

$${\text{D-score}}_{\text{PSD}}=\;\left\{\begin{array}{c} \sum_{d\in D}\sum_{p\in P}phase(d,p)\;\\ 0,D=\emptyset \end{array},\right.$$

where *D* is a set of selected diseases, and if *D* is empty, *D-score_PSD_* = 0. *P* is a set of all known phases for each disease. *Phase*(*p*,*d*) equals the phase number if there are known clinical trials for the selected disease in this phase and zero otherwise.

The clinical validity score reflects the number of the highest clinical trials phase (from 1 to 4) on which the drug was ever tested for any pathology.

#### 4.6.6. Method for Prediction of Pharmaceutical Compounds

In this study, the focus was on compounds with high pharmacological efficiency and low toxicity. For this purpose, a comprehensive library of chemical compounds and drugs was subjected to a SAR/QSAR analysis. This library contained 13,040 compounds with their pre-calculated potential pharmacological activities, their possible side and toxic effects, and possible mechanisms of action. All biological activities are expressed as probability values for a substance to exert this activity (Pa).

We selected compounds that satisfied the following conditions:

(i) Toxicity below a chosen toxicity threshold (defined as Pa, probability to be active as a toxic substance).
(ii) For all predicted pharmacological effects that correspond to a set of user-selected disease(s), Pa is greater than a chosen effect threshold.
(iii) There are at least 2 targets (corresponding to the predicted activity mechanisms) with predicted Pa greater than a chosen target threshold.

The maximum Pa value for all toxicities corresponding to the given compound is selected as the “Toxicity score.” The maximum Pa value for all activities corresponding to selected diseases for a given compound is used as the “Disease activity score.” The “Target activity score” (*T-score*) is calculated as follows:

$$\text{T-score}(s)=\frac{\left|T\right|}{\begin{array}{c} \left|T\right|*\omega (\left|AT\right|-\left|T\right|))\; \end{array}}\;\sum_{\begin{array}{c} m\in M(s) \end{array}}\left(pa\left(m\right)\sum_{g\in G(m)}IAP\left(g\right)optWeight(g)\right),$$

where *M*(*s*) is the set of activity mechanisms for the given structure (which passed the chosen threshold for activity mechanisms Pa). *G*(*m*) is the set of targets (converted to genes) that corresponds to a given activity mechanism (*m*) for a given compound. *pa*(*m*) is the probability of activity of the activity mechanism (*m*). *IAP*(*g*) is the invariant accuracy of prediction for a gene from *G*(*m*). *optWeight*(*g*) is the additional weight multiplier for a gene. *T* is set of all targets related to the compound intersecting the input list. |*T*| is number of elements in *T*. *AT* and |*AT*| are sets of all targets related to the compound and the number of elements in it. *w* is a weight multiplier.

The “Druggability score” (*D-score*) is calculated as follows:

$$\text{D-score}(g)=\mathrm{IAP}(g)\;\sum_{s\in S(g)}\sum_{m\in M(s,g)}pa(m),$$

where *S*(*g*) is the set of structures for which a target list contains a given target. *M*(*s*,*g*) is the set of activity mechanisms (for the given structure) that corresponds to the given gene. *pa*(*m*) is the probability of activity of the activity mechanism (*m*). *IAP*(*g*) is the invariant accuracy of prediction for a given gene.

## 5. Conclusions

We conducted an integrated multi-omic analysis combining transcriptomics and proteomics in a mouse model of tacrolimus-induced chronic nephrotoxicity. Through in silico upstream regulatory network analysis, PRMT1 and integrin αVβ6 emerged as potential therapeutic targets. To our knowledge, this is the first report to propose a possible mechanistic link between PRMT1 and integrins. These findings provide a conceptual framework for understanding the molecular pathogenesis of TACN and lay the groundwork for future studies involving validation in human samples and interventional experiments targeting PRMT1 and integrins in preclinical models.

## Figures and Tables

**Table 1:** Transcription factors potentially regulating differentially upregulated genes in TAC vs. control.

Gene Symbol	Gene Description	Regulatory Score ^1^	Yes–No Ratio ^2^
*HNF4A*	Hepatocyte nuclear factor 4 alpha	3.75	1.51
*STAT3*	Signal transducer and activator of transcription 3	3.33	1.87
*LEF1*	Lymphoid enhancer-binding factor 1	2.43	1.67
*SOX9*	SRY-box transcription factor 9	2.36	1.29
*NFATC1*	Nuclear factor of activated T cells 1	2.21	3.61
*NR2F1*	Nuclear receptor subfamily 2 group F member 1	2.17	5
*EOMES*	Eomesodermin	1.9	5.83
*TFCP2*	Transcription factor CP2	1.73	2.27
*EN1*	Engrailed homeobox 1	1.25	2.31
*MZF1*	Myeloid zinc finger 1	0.33	2.42
*MEIS1*	Meis homebox 1	0	8.33

^1^ The regulatory score is a measure of involvement of a given TF controlling the expression of genes that encode master regulators through positive feedback loops. ^2^ The Yes–No ratio is the ratio between frequencies of sites in Yes sequences versus those in No sequences. It describes the level of enrichment of binding sites for the indicated TF in regulatory target regions.

**Table 2:** Transcription factors potentially regulating differentially expressed downregulated genes in TAC vs. control.

Gene Symbol	Gene Description	Regulatory Score ^1^	Yes–No Ratio ^2^
*HSF1*	Heat shock transcription factor 1	2.57	2.75
*SMAD3*	SMAD family member 3	2.51	1.2
*SMAD2*	SMAD family member 2	2.36	1.4
*SMAD5*	SMAD family member 5	2.28	1.26
*SMAD1*	SMAD family member 1	2.21	1.2
*SOX9*	SRY-box transcription factor 9	2.1	3.34
*SMAD4*	SMAD family member 4	2.08	1.2
*SMAD9*	SMAD family member 9	1.98	1.2
*SMAD7*	SMAD family member 7	1.94	1.15
*SMAD6*	SMAD family member 6	1.78	1.2
*FLI1*	Fli-1 proto-oncogene, ETS transcription factor	1.67	2.63
*HIC1*	HIC ZBTB transcriptional repressor 1	1.53	1.17
*VDR*	Vitamin D receptor	1.41	2.32
*LEF1*	Lymphoid enhancer-binding factor 1	1.41	10
*KLF4*	KLF transcription factor 4	1.35	1.89
*PAX2*	Paired box 2	1.25	1.52
*NKX2-5*	NK2 homeobox 5	0.97	1.63
*NFIC*	Nuclear factor I C	0.55	1.47
*FIGLA*	Folliculogenesis-specific bHLH transcription factor	0	1.15

^1^ The regulatory score is a measure of involvement of a given TF in controlling expression of genes that encode master regulators via positive feedback loops. ^2^ The Yes–No ratio is the ratio between frequencies of sites in Yes sequences versus No sequences. It describes the level of enrichment of binding sites for the indicated TF in regulatory target regions.

**Table 3:** The top ten master regulators of upregulated genes in TAC vs. control.

Master Molecule Name	Gene Symbol	Gene Description	Total Rank ^1^	Log FC (Transcriptome)	Log FC (Proteome)
HRMT1L2(h)	*PRMT1*	Protein arginine methyltransferase 1	94	0.43	
Integrins	*ITGA1*, *ITGA2B*, *ITGA3*, *ITGA4*, *ITGA5*, *ITGA6*, *ITGA8*, *ITGA9*, *ITGAL*, *ITGAV*, *ITGB1*, *ITGB2*, *ITGB3*, *ITGB4*	Integrin subunit alpha 1, integrin subunit alpha 2b, integrin subunit alpha 3	95	1.57	0.43
PTPRD(h)	*PTPRD*	Protein tyrosine phosphatase receptor type D	154	0.4	
STAT3(h)	*STAT3*	Signal transducer and activator of transcription 3	167	0.25	0.34
Cdk4-isoform1(h): cyclinD1a(h)	*CCND1*, *CDK4*	Cyclin D1, Cyclin-dependent kinase 4	176	0.47	0.38
HRMT1L2-isoform2(h)	*PRMT1*	Protein arginine methyltransferase 1	182	0.43	
HRMT1L2-isoform4(h)	*PRMT1*	Protein arginine methyltransferase 1	182	0.43	
HRMT1L2-isoform1(h)	*PRMT1*	Protein arginine methyltransferase 1	184	0.43	
HRMT1L2-isoform3(h)	*PRMT1*	Protein arginine methyltransferase 1	184	0.43	
SIRT2(h)	*SIRT2*	Sirtuin 2	198	0.27	

^1^ Total rank is the sum of ranks of master molecules sorted by key node score, CMA score, transcriptomics, and proteomic data. The blank in the table denotes ‘not applicable’.

**Table 4:** Top ten master regulators potentially governing regulation of downregulated genes in TAC vs. control.

Master Molecule Name	Gene Symbol	Gene Description	Total Rank ^1^	Log FC (Transcriptome)	Log FC (Proteome)
Ubc9(h)sumo3C93: sumo3(h){clCG92,93}	*SUMO3*, *UBE2I*	Small ubiquitin-like modifier 3, Ubiquitin-conjugating enzyme E2 I	55	−0.62	
Ubc9{sumo3C93}: sumo3{clCG92,93}	*SUMO3*, *UBE2I*	Small ubiquitin-like modifier 3, Ubiquitin-conjugating enzyme E2 I	81	−0.62	
M-CSF-1-R(h)	*CSF1R*	Colony-stimulating factor 1receptor	99	−0.48	
M-CSF-1-R-isoform2(h)	*CSF1R*	Colony-stimulating factor 1receptor	109	−0.48	
Aos1(h): SAE2(h)sumo3C173: sumo3(h){clCG92,173}	*SAE1*, *SUMO3*, *UBA2*	SUMO1-activating enzyme subunit 1, small ubiquitin-like modifier 3, ubiquitin-like modifier-activating enzyme 2	140	−0.62	
MKP3(h)	*DUSP6*	Dual-specificity phosphatase 6	168	−0.27	
Siah1(h)	*SIAH1*	Siah E3 ubiquitin protein ligase 1	169	−0.44	
Merlin(h)	*NF2*	NF2, moesin–ezrin–radixin-like (MERLIN) tumor suppressor	181	−0.34	
Ubc6(h)	*UBE2J1*	Ubiquitin-conjugating enzyme E2 J1	184	−0.47	
Hsp70-1(h)	*HSPA1A*	Heat shock protein family A(Hsp70) member 1A	197	−2.96	

^1^ Total rank is the sum of ranks of master molecules sorted by key node score, CMA score, transcriptomic and proteomic data. The blank in the table denotes ‘not applicable’.

**Table 5:** Prospective drug targets selected from the full list of identified master regulators, filtered by their Druggability scores derived from Human PSD^TM^.

Gene Symbol	Gene Description	Druggability Score	Total Rank ^1^	Log FC (Transcriptome)	Log FC (Proteome)
*PRMT1*	Protein arginine methyltransferase 1	2	94	0.43	
*ITGAL*	Integrin subunit alpha L	14	95	1.57	0.43
*ITGAV*	Integrin subunit alpha V	3	95	1.57	0.43
*STAT3*	Signal transducer and activator of transcription 3	33	167	0.25	0.34
*CCND1*	cyclin D1	45	176	0.47	0.38
*SIRT2*	Sirtuin 2	5	198	0.27	

^1^ Total rank is the sum of ranks of master molecules sorted by key node score, CMA score and expression change score (log FC, if present). A blank in the table represents ”not applicable”.

**Table 6:** Prospective drug targets selected from the full list of identified master regulators, filtered by their Druggability scores predicted using PASS software.

Gene Symbol	Gene Description	Druggability Score	Total Rank ^1^	Log FC (Transcriptome)	Log FC (Proteome)
*PRMT1*	Protein arginine methyltransferase 1	0.65	94	0.43	
*ITGAL*	Integrin subunit alpha L	7.77	95	1.57	0.43
*ITGB6*	Integrin subunit beta 6	5.31	95	1.57	0.43
*ITGAV*	Integrin subunit alpha V	5.31	95	1.57	0.43
*ITGA1*	Integrin subunit alpha 1	5.31	95	1.57	0.43
*PTPRD*	Protein tyrosine phosphatase receptor type D	17.02	154	0.4	

^1^ Total rank is the sum of ranks of master molecules sorted by key node score, CMA score and expression change score (log FC, if present). A blank in the table represents ”not applicable”.

## Data Availability

The datasets generated and/or analyzed during the present study are available from the corresponding author on request.

## Supplementary Materials

The supporting information can be downloaded at: https://www.mdpi.com/article/10.3390/ijms262110282/s1.

## Abbreviations

The following abbreviations are used in this manuscript:

ADMA	Asymmetric dimethyl arginine
CCND1	Cyclin D1
CDK4	Cyclin-dependent kinase 4
CKD	Chronic kidney disease
CMA	Composite Module Analyst
CSF1R	Colony-stimulating factor 1 receptor
DUSP6	Dual-specificity phosphatase 6
EN1	Engrailed homeobox 1
EOMES	Eomesodermin
FDRs	False discovery rates
FIGLA	Folliculogenesis-specific bHLH transcription factor
FLI1	Fli-1 proto-oncogene, ETS transcription factor
HIC1	HIC ZBTB transcriptional repressor 1
HNF4A	Hepatocyte nuclear factor 4 alpha
HSF1	Heat shock transcription factor 1
HSPA1A	Heat shock protein family A(Hsp70) member 1A
KLF4	KLF transcription factor 4
LEF1	Lymphoid enhancer-binding factor 1
MEIS1	Meis homebox 1
MZF1	Myeloid zinc finger 1
NAD^+^	Nicotinamide adenine dinucleotide
NF2	NF2, moesin–ezrin–radixin-like (MERLIN) tumor suppressor
NFATC1	Nuclear factor of activated T cells 1
NFIC	Nuclear factor I C
NKX2-5	NK2 homeobox 5
NO	Nitric oxide
NOS	Nitric oxide synthase
NR2F1	Nuclear receptor subfamily 2 group F member 1
PAX2	Paired box 2
PRMT1	Protein arginine methyltransferase-1
PTPRD	Protein tyrosine phosphatasereceptor type D
PWMs	Position weight matrices
SAE1,	SUMO1-activating enzyme subunit 1
SIAH1	Siah E3 ubiquitin proteinligase 1
SIRT2	Sirtuin 2
SOX9	SRY-box transcription factor 9
STAT3	Signal transducer and activator of transcription 3
SUMO3,	Small ubiquitin-like modifier 3,
TAC	Tacrolimus
TACN	Tacrolimus-induced chronic nephrotoxicity
TFs	Transcription factors
TFBSs	Transcription factor binding sites
TFCP2	Transcription factor CP2
UBA2	Ubiquitin-like modifier-activating enzyme 2
UBE2J1	Ubiquitin-conjugating enzyme E2 J1
VDR	Vitamin D receptor
