# Supplementary material for: Trans-Omic Analysis Identifies the ‘PRMT1–STAT3–Integrin αVβ6 Axis’ as a Novel Therapeutic Target in Tacrolimus-Induced Chronic Nephrotoxicity
Source: Int J Mol Sci. 2025 Oct 22;26(21):10282. doi: 10.3390/ijms262110282 (PMC12607680; doi:10.3390/ijms262110282)
Supplement: Supplementary file 1 [file ijms-26-10282-s001.zip › ijms-3874714-supplementary.pdf]

Supplementary information for

Trans-Omic Analysis Identifies the 'PRMT1–STAT3–Integrin αVβ6 Axis' as a Novel Therapeutic Target in Tacrolimus-Induced Chronic Nephrotoxicity

Sho Nishida [1,2], Tamaki Ishima [1], Daiki Iwami [2], Ryozo Nagai [3] and Kenichi Aizawa [1, 4*]

[1] Department of Translational Research, Clinical Research Center, Jichi Medical University Hospital, Shimotsuke 329-0498, Japan
[2] Division of Renal Surgery and Transplantation, Department of Urology, Jichi Medical University, Shimotsuke 329-0498, Japan
[3] Jichi Medical University, Shimotsuke 329-0498, Japan
[4] Clinical Pharmacology Center, Jichi Medical University Hospital, Shimotsuke 329-0498, Japan
* Correspondence: aizawa@jichi.ac.jp

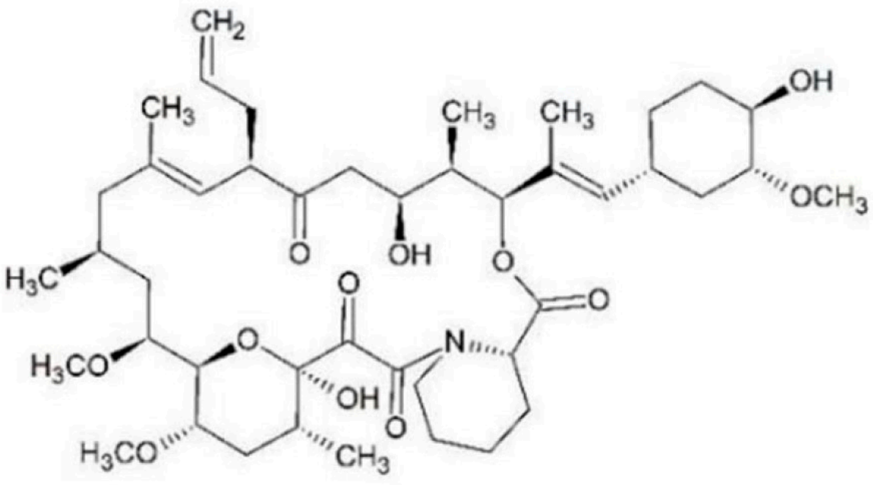

Supplemental Figure S1. The molecular structure of tacrolimus (TAC). TAC inhibits calcineurin, which is involved in T-cell activation and proliferation. This inhibition prevents the transcription of interleukin-2, thereby suppressing immune responses. The figure is adapted from Aizawa et al., (*Int J Mol Sci*. 2025; 26(13):6358; https://doi.org/10.3390/ijms26136358).

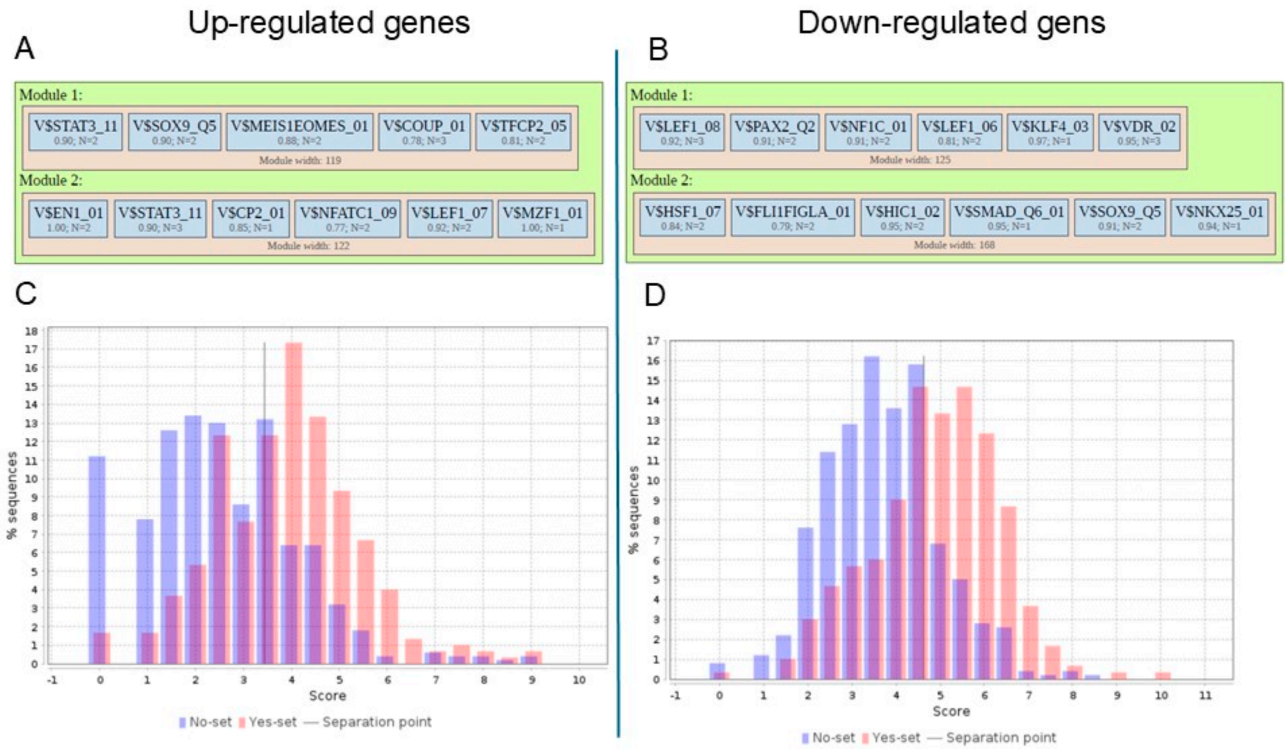

Supplemental Figure S2. Enhancer models potentially involved in regulation of target genes.

To build the most specific composite modules we choose top 300 significant both up-regulated genes and down-regulated genes as the input of CMA algorithm. The obtained CMA model is then applied to compute CMA score for all up-regulated genes and down-regulated genes in TAC vs. Control. (A-B)-the most specific composite modules obtained as the results of CMA analysis. 'Module width' represent the preferable distance between sites. The PWM name is presented in the upper part of light blue rectangle. "V$" means "vertebrates"; the TF family name shows at the right part of PWM name. Score of the best match is shown in the lower part of the light rectangle as the optimized cut-off of the PWM score; number of individual matches (N) for each PWM gives the maximal number of TF sites with the highest scores, which are computed in the module. (C-D)- two histograms are shown here and represent the distribution of model scores for up-regulated genes and down regulated genes. Blue bars show No-set genes (unchanged genes). Orange bar represents YES-set genes (changed genes). AUC =0.74 for up-regulated genes and 0.73 for down-regulated genes.

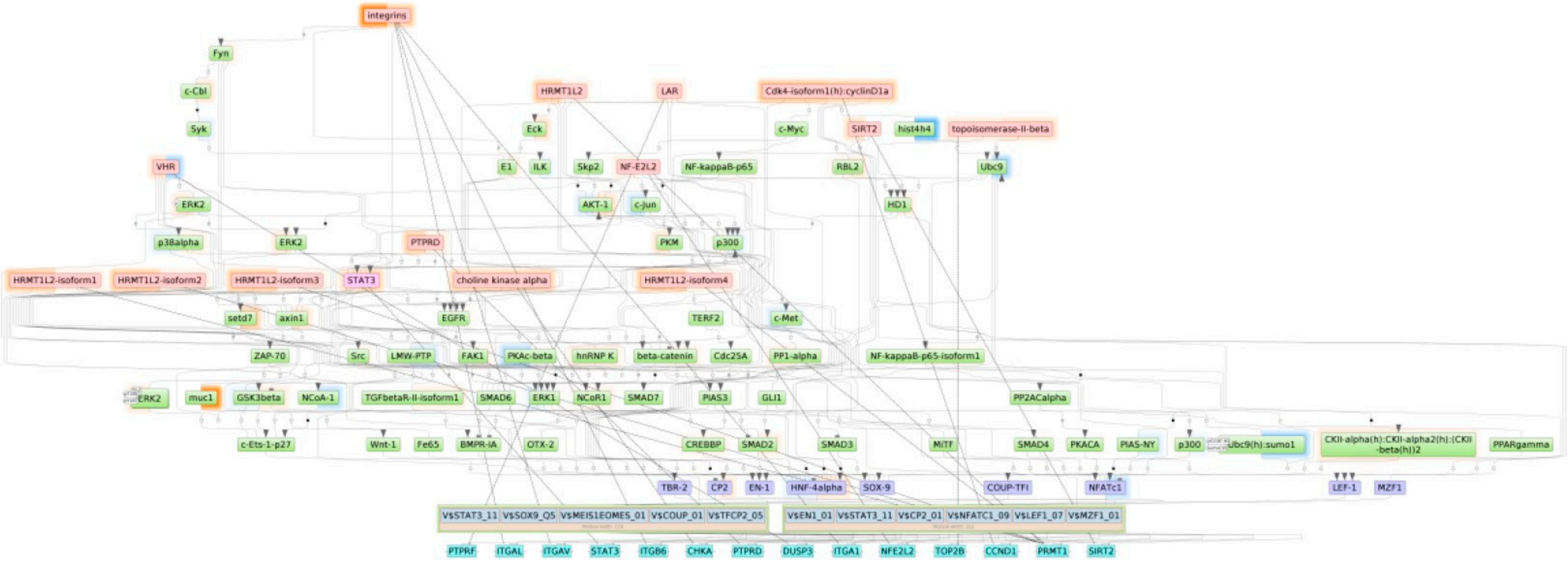

Supplemental Figure S3. Diagram of intracellular regulatory signal transduction pathways of up-regulated genes in TAC vs. Control. Master regulators are indicated with red rectangles, transcription factors are purple rectangles, and green rectangles are intermediate molecules, which have been added to the network during research for master regulators from selected TFs. Orange and blue frames highlight molecules that are encoded by up- and downregulated genes. The left half of a highlighting frame corresponds to transcriptomic data, the right one to proteomic data. Positive feedbacks are represented by dotted lines

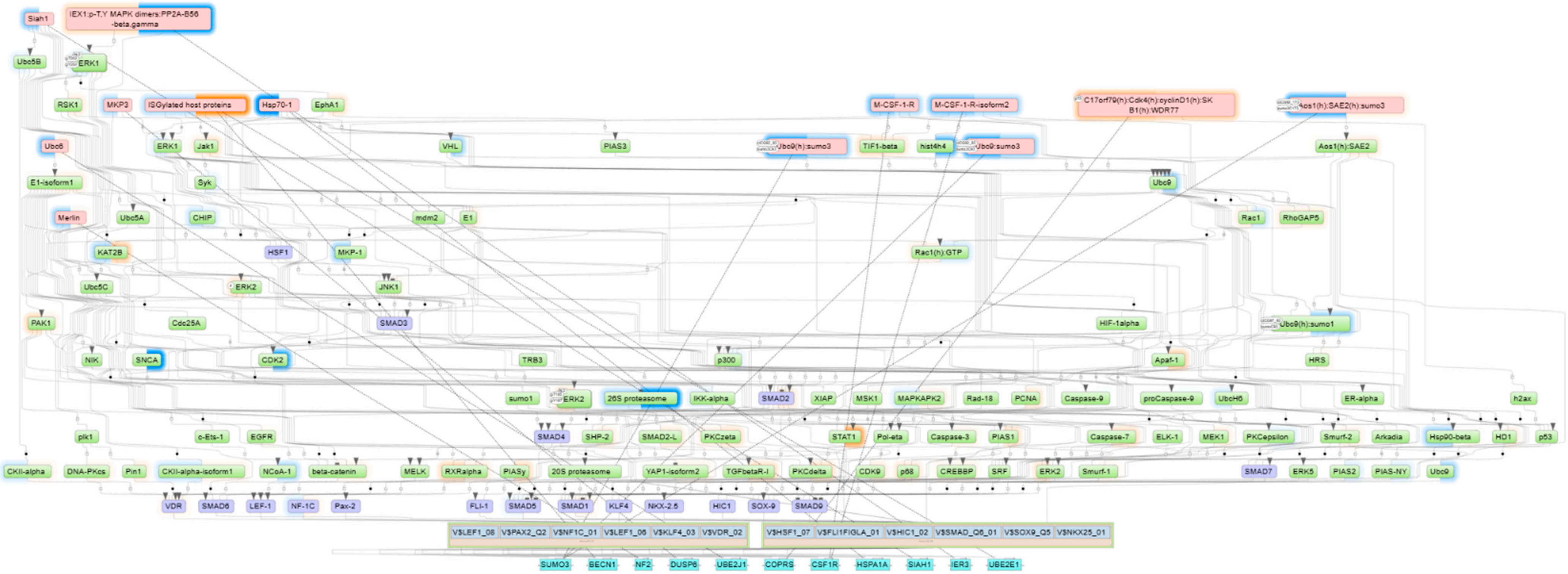

Supplemental Figure S4. Diagram of intracellular regulatory signal transduction pathways of down-regulated genes in TAC vs. Control. Master regulators are indicated with rectangles, transcription factors are purple rectangles, and green rectangles are intermediate molecules, which have been added to the network during research for master regulators from selected TFs. Orange and blue frames highlight molecules that are encoded by up- and downregulated genes. The left half of a highlighting frame corresponds to transcriptomic data, the right one to proteomic data. Positive feedbacks are represented by dotted lines.
